# C-terminus–independent multimerization of Hv1 proton channels

**DOI:** 10.1016/j.jbc.2026.113387

**Published:** 2026-08-04

**Authors:** Victor de la Rosa, Gisela E. Rangel-Yescas, Oscar Flores-Herrera, Mercedes Esparza-Perusquia, Gema R. Cristóbal-Mondragón, I. Scott Ramsey, Tamara Rosenbaum, León D. Islas

**Affiliations:** 1 Departamento de Fisiología, Facultad de Medicina, UNAM, Mexico City, Mexico; 2 Departamento de Bioquímica, Facultad de Medicina, UNAM, Mexico City, Mexico; 3 Department of Physiology and Biophysics, Virginia Commonwealth University, Richmond, Virginia, USA; 4 Departamento de Neurociencia Cognitiva, Instituto de Fisiología Celular, UNAM, Mexico City, Mexico

**Keywords:** Voltage-gated proton channels, protein dimerization, channel gating, activation

## Abstract

The voltage-gated proton channel Hv1, assembles as a dimer when expressed in heterologous systems, likely reflecting its stoichiometry in native membranes. It is known that the dimeric interaction is mediated at least by specific transmembrane domain amino acid interactions and a coiled-coil interaction mediated by the intracellular C-terminus helices. Deletion of this last interaction (ΔC) has been thought to lead to disruption of dimers and production of monomeric channels, which remain functional as proton channels, albeit with different biophysical properties. Here, we present evidence that apart from activation kinetics, other biophysical properties of the ΔC channels are different to those of the WT, dimeric channels, and that the ΔC channels are still capable of efficiently forming dimers and maybe even reaching higher stoichiometry. Our results suggest that the C-terminal-mediated interactions might help to ensure formation of dimers in full-length channels but are not necessary for their assembly.

Voltage-gated proton-selective Hv1 channels are important regulators of intracellular pH and play several other important roles in cellular physiology and pathophysiology. These channels are expressed in almost all cell types and found in all eukaryotes (1, 2, 3). In most organisms, Hv1 channels are present in the plasma membrane; however, they are also present in intracellular membranes of others, such as unicellular coccolithophores and dinoflagellates, where they play roles in cellular excitability and calcification (4, 5).

Although not all physiological roles of Hv1 have been uncovered, some basic biophysical properties are relatively well established. The building block of Hv1 is the membrane protein fold known as a voltage-sensing domain (VSD). This domain is constituted by four antiparallel alpha helices that form a bundle through highly specific interactions. The fourth helix contains a canonical sequence of positively charged amino acids found in all voltage sensing domains, although with different number of charged residues, followed by a long intracellular alpha helix predicted to be involved in coiled-coil interactions.

Upon depolarization, the S4 segment undergoes activating, voltage-induced transitions that are ultimately translated into proton permeation through the same protein domain. While voltage-sensitive transitions in Hv1 seem to follow some of the same principles as in other VSDs (6, 7), proton permeation is much less well understood (8, 9). One characteristic of Hv1 channels that seems to be well established, is that the functional form of the channel in a membrane environment is as a homodimer (10, 11, 12, 13), a conclusion that was put forward based on biochemical and functional experiments in the human Hv1 (hHv1) channel and the *Ciona intestinalis* homolog. Dimerization is proposed to be necessary for voltage-dependent gating cooperativity and to produce the sigmoidal activation of ionic currents (14). It is generally accepted that monomerized Hv1 conductance is half that of the dimer, although it has not been established whether expressed Hv1-ΔC channels generate current as monomers or whether dimerization of truncated channels is necessary for their function. It has been shown that the main regulator of this dimeric interaction is the long C-terminal alpha helix, which forms a coiled-coil structure (15, 16).

However, there is evidence that a single point mutation in the S2 segment (E153) also disrupts cooperativity and shifts the voltage-dependence of gating to negative voltages (17). It has also been shown that other dimer-mediating interactions are possible through transmembrane domains. One such interaction was initially suggested to involve contacts between residues in the S1 segment (13). More recently interactions between S1 and S4 segments have been elucidated, constraining the organization of the two subunits within the dimer (11). One question that arises is then to what degree coiled-coil and transmembrane domain interactions are capable of maintaining a dimeric interaction? Are both necessary and sufficient or could dimers be stabilized by either?

To begin to answer these questions, we have applied FRET and patch-fluorometry approaches to investigate the oligomeric state of Hv1 channels and the contribution of the coiled-coil to its maintenance.

## Results

One of the characteristics of hHv1 channels is that their subunits are organized in a dimer. This is the functional form found when the channel is expressed in a heterologous expression system (12, 13). Also, the *C. intestinalis* Hv1 seems to form dimers in expression systems (15). We do not know if this is the form found in native tissues expressing Hv1 proton channels, but this is likely to be the case, as has been shown in human neutrophils (18). It has been previously demonstrated that dimerization is strongly dependent on the formation of a coiled-coil structure by the C-terminal alpha helices (16), although a second dimeric interaction surface was proposed to be formed by some external S1 residues (13).

**Figure 1:**
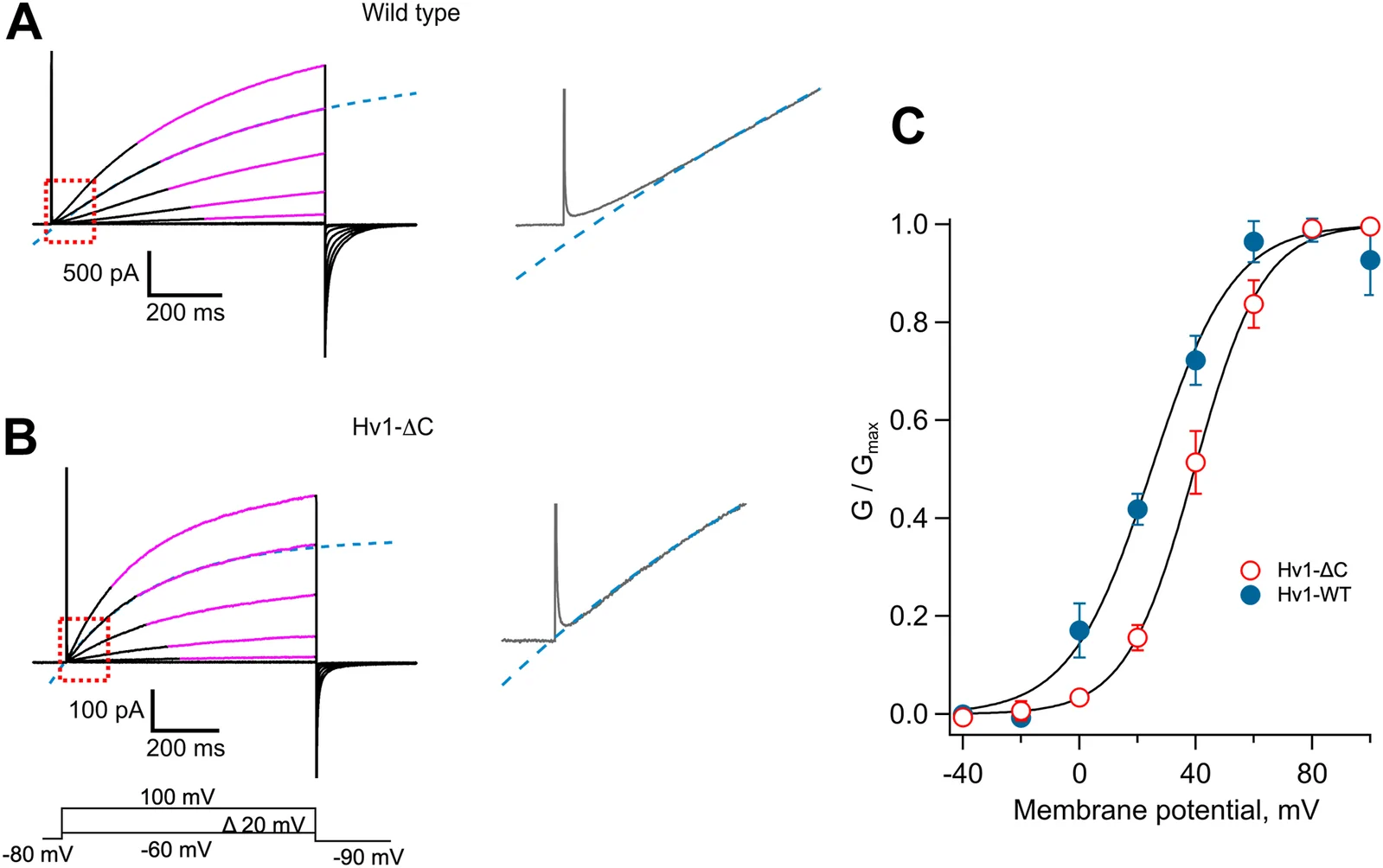
**Gating differences between WT and ΔC channels expressed in *Xenopus* oocytes.***A*, representative current families of WT and (*B*) ΔC channels. WT channels show a prominent delay. The *dotted blue curve* is the exponential fit to Equation 3 as indicated in the methods section. The second half of current activation used for the exponential fit is shown in *purple* for all traces. The panels to the right show that the delay in activation disappears in ΔC channels, since the exponential function extrapolates to the start of the voltage pulse. The voltage protocol used for currents in A and B is shown at the bottom of the figure. *C*, normalized conductance of both types of channels. The *black curves* are the fits of the data to Equation 2 with parameters: q = 1.79 e_o_, V_0.5_ = 23.9 mV for WT and for ΔC: q = 2.13 e_o_, V_0.5_ = 39.6 mV. Data points are the mean of n = 5 independent patches. Error bars are the SD The mean value of the V_0.5_ for WT is 24.0 mV and for ΔC is 40.7 mV, which are statistically different (*t* test, *p* = 3.90 x 10^−5^). Both expressed channels are N-terminal tagged with mVenus fluorescent protein.

Deletion of the carboxy-terminus (K222-Stop), yielding channels labeled as Hv1-ΔC, was shown to be sufficient to reduce the co-immunoprecipitation of differentially tagged Hv1-ΔC, but not completely prevent it (10). As a functional consequence of this deletion, Hv1-ΔC channels activate at more positive potentials (Fig. 1*C*) and faster than WT channels. They also lose the sigmoidal activation characteristic of WT-Hv1 and show a disappearance of the activation delay in response to a depolarizing voltage pulse (Fig. 1*B*). This delay, typical of channels with sigmoidal activation can be quantified in several ways. Figure 1*A* shows that when an exponential function with a time delay term, δ, (Equation 3) is fitted to the second half of the activation time course of WT channels, it extrapolates to a time after the beginning of the depolarizing voltage pulse given by δ. In contrast, in Hv1-ΔC channels, the exponential extrapolates essentially to the beginning of the voltage pulse, indicating the disappearance of the delay and sigmoidal activation (Fig. 1*B*). It has been proposed that dimeric channels gate cooperatively and that this cooperativity is evidenced by the sigmoidal activation and a larger apparent gating charge of WT channels, when compared to monomeric, ΔC channels (15). These observations have been taken as hallmarks of monomeric and dimeric channels.

Apart from the sigmoidal activation time course, what other biophysical properties are affected by deletion of the C-terminus of hHv1 channels? Since measurement of the functionality of Hv1 channels at the single channel level is extremely difficult, we applied a patch-clamp fluorometry approach to study the function of monomeric and dimeric channels. Since each protomer is tagged with a single fluorescent protein, each protomer should contribute the same fluorescence intensity to the total fluorescence signal. The function of channels can be measured simultaneously with fluorescence by recording proton currents from the same patch. Under these conditions, macroscopic current *I*, at a specified voltage, *V*, is given by:

I(V)=N·Po(V)·i(V)

where *Po(V)* and *i(V)* are the open probability and the single-channel current at voltage *V* and *N* is the number of channels. For fluorescently tagged channels, *N ∼ F*, where *F* is the fluorescence intensity.

According to the proposal that ΔC channels are functional monomers and full-length channels are dimers, it is expected that dimers will have double the fluorescence intensity and double the single channel current as monomeric ΔC channels. Thus, a plot of *I versus F* for a sufficiently depolarized voltage where *Po* is maximal, should have the same slope for both channels and the slope should be proportional to *i*. A similar approach has been previously applied to estimate the single channel current size of CNG and HCN channels (19).

**Figure 2:**
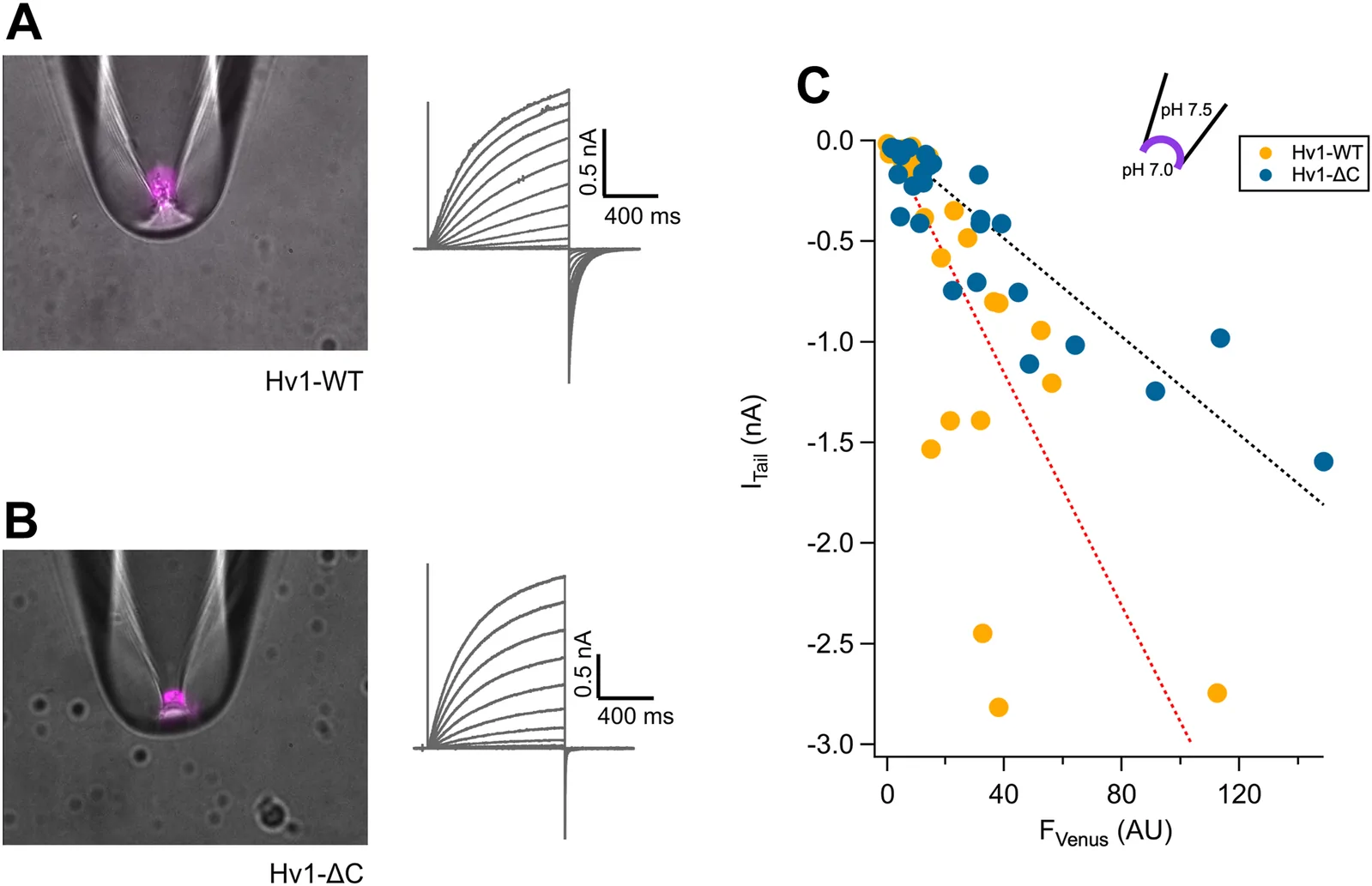
**Different activity levels in WT and ΔC human Hv1channels.***A*, image of a patch pipette superimposed on the fluorescence image of an inside-out patch containing WT channels tagged with the mVenus fluorescent protein. Next to the image is a family of proton currents recorded simultaneously. *B*, similar to (*A*) for ΔC channels. *C*, the tail current recorded at −80 mV from pulses at +80 mV and +100 mV for WT and ΔC, respectively, was plotted as a function of the fluorescence of the patch. Linear functions were least-squares fitted to the data. Correlation coefficients are r = 0.748 for WT and r = 0.888 for ΔC. The inset indicates the proton gradient used for these recordings.

Proton currents and fluorescence intensity were recorded in inside-out patches from *Xenopus* oocytes expressing both WT (Fig. 2*A*) and ΔC channels (Fig. 2*B*). The patch fluorescence was recorded at the beginning of the experiment and mostly corresponds to the area of membrane that is under voltage-clamp. Recording conditions were such that the ΔpH = 0.5. Tail currents, I_tail_, were measured from patches containing WT mVenus-Hv1 or mVenus-Hv1-ΔC (Fig. 2*C*) at equivalent *Po* (+80 mV and +100 mV, respectively, see Fig. 1*A*). I_tail_, was chosen since it is faster and measured at a fixed negative voltage, making it less prone to be affected by proton depletion (20). mVenus fluorescence is expected to be directly proportional to channel subunit number (*N*). If monomeric and dimeric Hv1 channels function equivalently, I_tail_ *versus* F_Venus_ is expected to be the same (*i.e.*, *i*_*dimer*_ *= i*_*monomer*_). The data show that the I *versus* F relationships for both types of channels have a different slope (Fig. 2*C*). The observed difference in I_tail_ vs. F_Venus_ suggests that compared to WT, proportionally fewer Hv1-ΔC channels generate current.

These results suggest that truncation of the C-terminus in hHv1 channels does more than just interrupt the formation of dimers and instead leads to reduced channel activity relative to expression, possibly through reduced single-channel conductance, open probability or both.

To estimate the oligomeric state of hHv1 channels, we carried out FRET measurements between subunits tagged with fluorescent proteins. FRET measurements have been previously carried out in the *C. intestinalis*, CiHv1-WT and ΔNΔC-channels, which have a double truncation of the N-terminus and C-terminus and which were deemed to be monomeric (15). Those experiments were limited in scope and for that reason, we decided to systematically measure the multimerization state in WT and ΔC hHv1 *in vivo* using FRET between fluorescent proteins attached to the channels. We employed monomeric versions of Citrine and Cerulean as acceptor and donor, respectively and measured FRET with a spectroscopic approach, which considers the combinatorics of channel formation (see methods).

**Figure 3:**
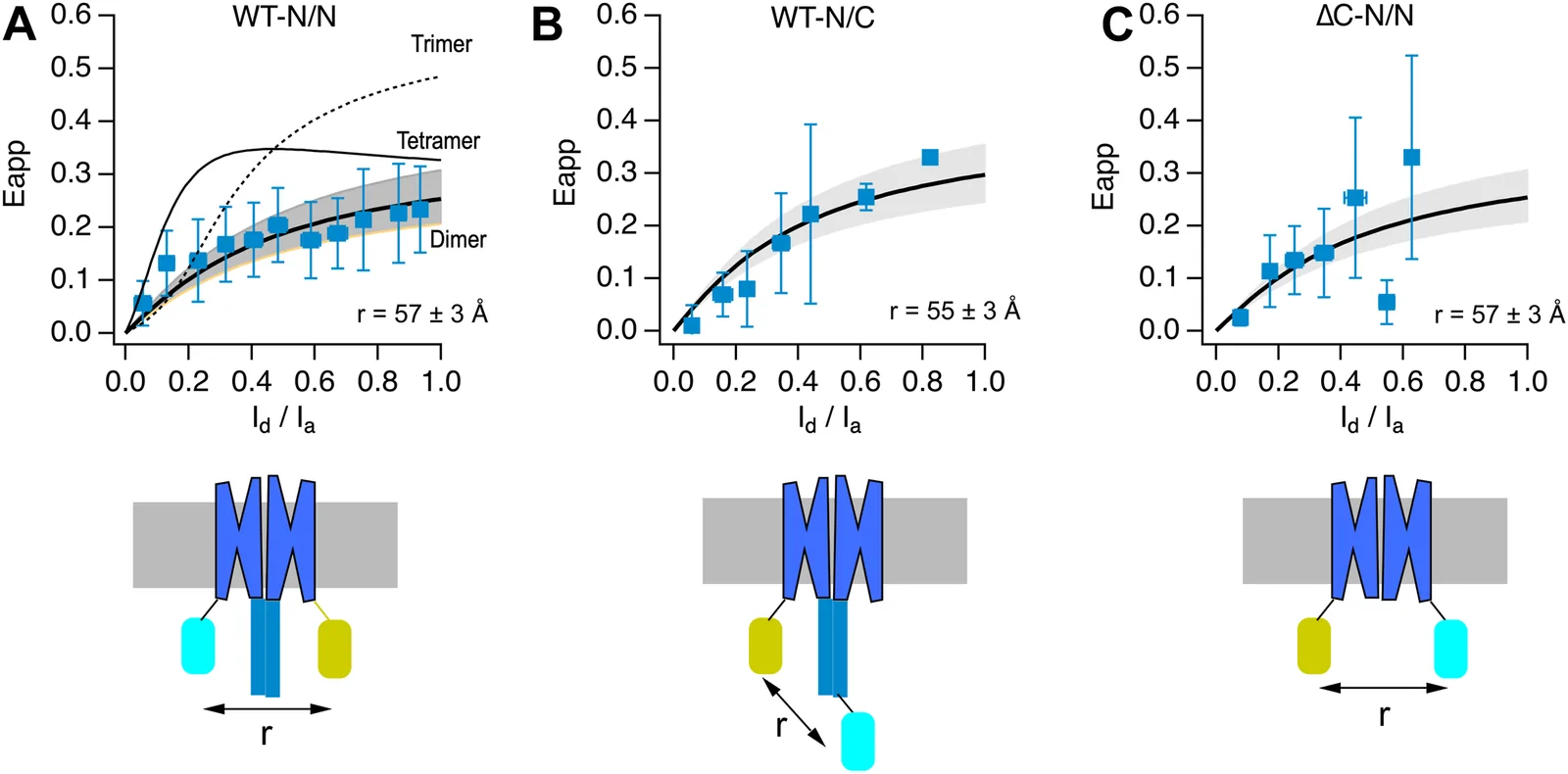
**Experimental detection of formation of dimeric channels in WT and ΔC channels by FRET.***A*, apparent FRET efficiency as a function of the donor-acceptor ratio I_d_/I_a_ measured from channels tagged with mCerulean and mCitrine at the N terminus. The *blue squares* and error bars are the mean and SD from measurements of 203 individual cells. The *dark curve* is the prediction of Equations 5 and 6, for a dimer with fluorophores separated by the indicated distance *r*, with a distance dispersion indicated by the ± value. The *dotted curve* is the prediction for a trimer and the *thin solid curve* the prediction for a tetramer, both with the same separation of 57 Å between fluorophores. *B*, WT dimers formed between subunits tagged in the N-terminus and C-terminus. *C*, dimers can be formed between ΔC subunits. Robust FRET is observed that is also consistent with a dimer. In all panes, the distance dispersion, *A*, predicts the FRET efficiency dispersion indicated by the *shaded region*. Below each data panel is a cartoon depicting the expected organization of the dimers and FPs in each condition. Data points and errors are the mean ± SD.

As has been shown before, employing biochemical approaches, WT hHv1 forms dimers (13). In our experiments, channels were tagged at the N-terminus to reduce the possibility of the fluorescent protein interfering with dimer formation mediated by the C-terminus. We can readily detect oligomer formation by FRET (Fig. 3*A*). The FRET efficiency as a function of donor/acceptor abundance, can be fit to different geometric models of oligomerization. Figure 3*A* compares the predicted FRET for a dimer, trimer or a tetramer with the same distance between monomers and shows that the most likely oligomerization state is dimeric. We also demonstrate that dimer formation in WT channels is not dependent on the position of the fluorescent proteins. Dimers are readily formed between WT channels tagged in the N- and the C-termini, independently (Fig. 3*B*).

Contrary to expectation, we also detected robust FRET efficiency between N-terminal tagged subunits of C-terminus deleted channels (Fig. 3*C*), consistent with dimer formation.

**Figure 4:**
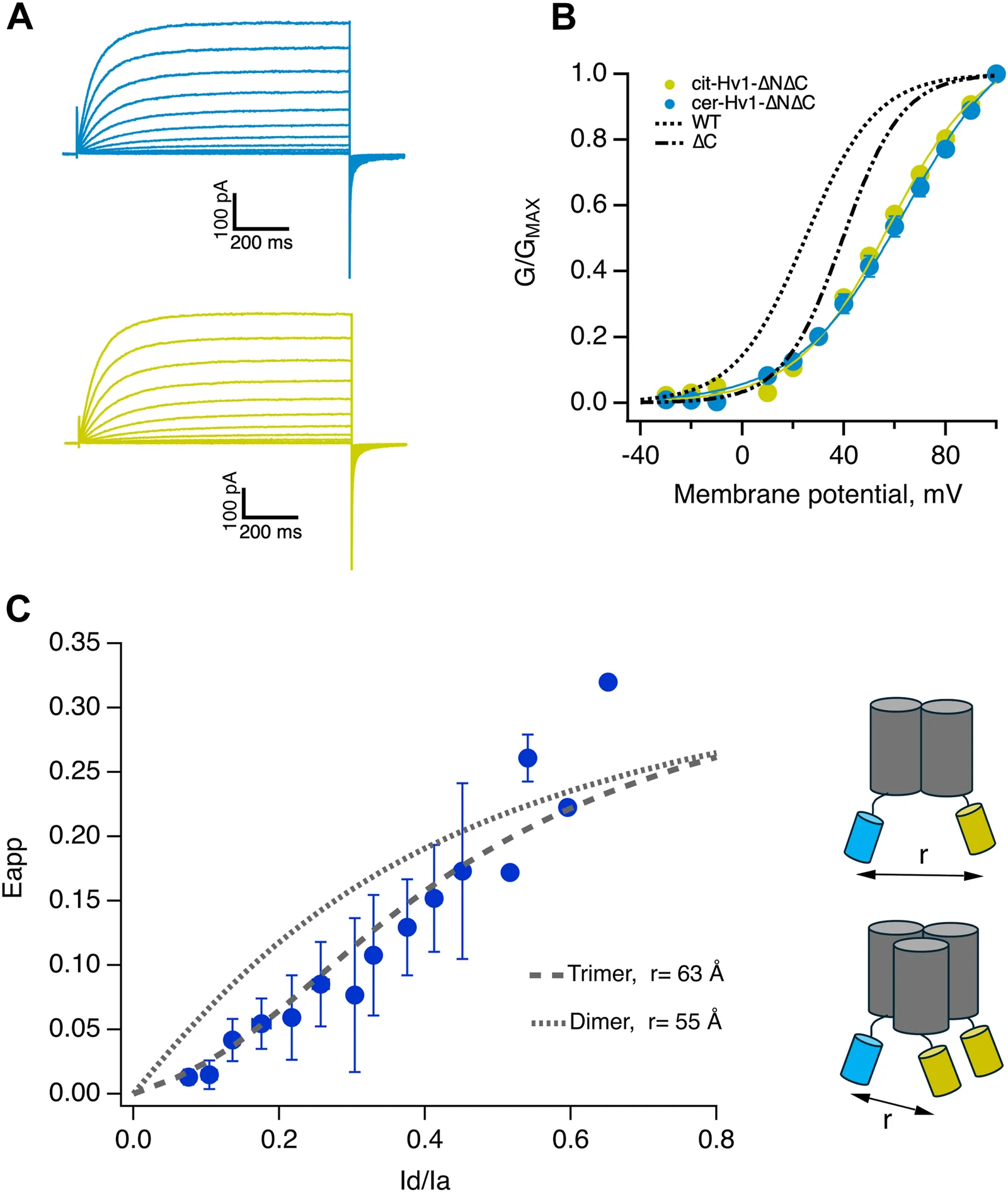
**FRET in the N- and C-truncated hHv1 channel.***A*, functional expression of the ΔNΔC-hHv1 channels tagged with mCitrine or mCerulean in the N-terminus used to measure FRET. *B*, the G-V curves of these constructs compared to WT and ΔC channels (*dotted* and *dashed curves* as indicated in the figure). The mean value of the G-V curves of the ΔNΔC are indicated by *colored circles*. The error bars are the SD, n = 5 patches for each construct. *C*, apparent FRET efficiency is shown in the *left panel*. Symbols are the mean and the error bars represent SD; n = 98 independent cells. *Dotted* and *dashed curves* shown are the fits to dimer or trimer models with the main distance between FRET pairs indicated by *r* (*right panel*). The fit suggests that the most likely stoichiometry of this construct is as trimers in the membrane of HEK cells.

Since the previous FRET experiments with CiHv1 employed channels in which the N-terminus and C-terminus were simultaneously deleted (15), we constructed a similar human channel, ΔNΔC-hHv1 tagged with FPs in the N-terminus and assessed their interaction. These constructs also yield robust currents in inside-out patches from human embryonic kidney (HEK) cells which are faster and activate at more positive voltages than WT channels (Fig. 4, *A* and *B*). When coexpressed, these truncated channels generate FRET, indicating that these constructs lacking both intracellular domains can still interact. The stoichiometry of these channels is, however, more consistent with trimers according to our assembling model (Fig. 4*C*), although definitive assignment of stoichiometry will require additional experiments. Regardless, these observations indicate that Hv1 channels lacking intracellular domains are still capable of forming higher order oligomeric assemblies.

To reduce the possibility of interaction between fluorescent proteins, we used monomeric versions in all our experiments; however, we carried out a control experiment overexpressing two FP-tagged channels that should not interact (TRPV1 and hHv1) and did not detect any FRET, supporting the conclusion that FRET between Hv1 constructs is specific and not mediated by a spurious interaction between the FPs (Fig. S1).

**Figure 5:**
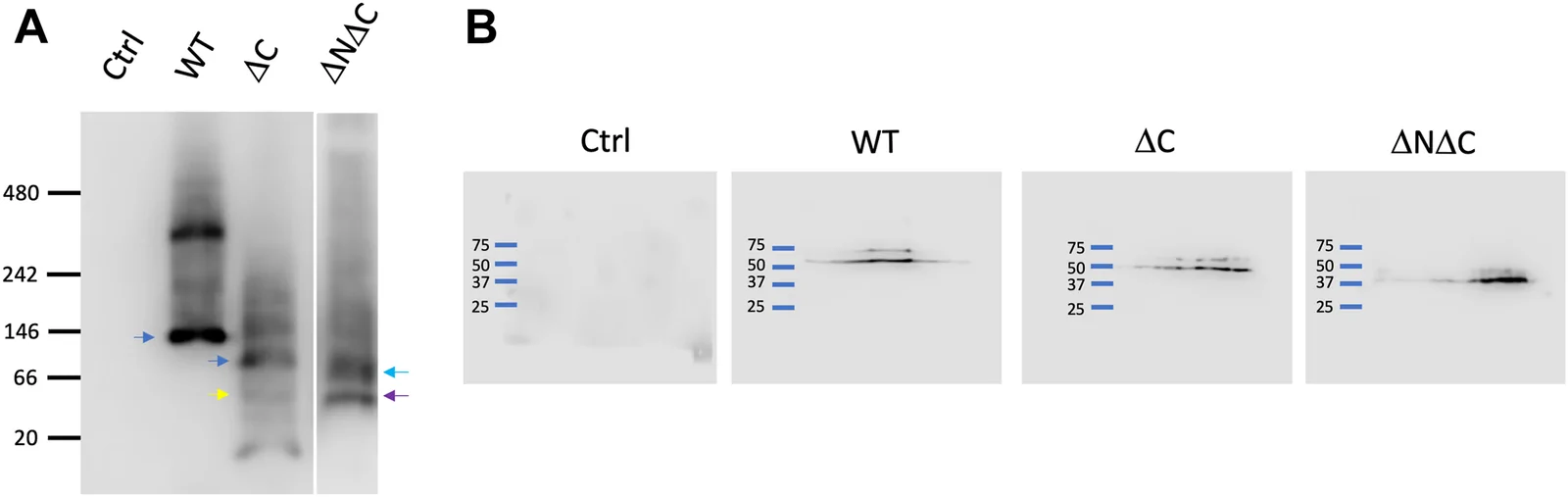
**Native gel of WT and constructs lacking C and C-and-N terminal domains.***A*, non-denaturing gel blotted against mCitrine with an anti-GFP antibody. The ΔNΔC lane was exposed three times longer. The construct is indicated at the top of each lane. *Blue arrows* indicate the expected molecular weight of dimers for each construct. WT-hHv1 122 kDa, hHv1-ΔC 102 kDa, and hHv1-ΔNΔC, 80 kDa. Expected molecular weights for the monomers are: WT-hHv1 61 kDa, hHv1-ΔC 51 kDa, and hHv1-ΔNΔC, 40 kDa. The *yellow arrow* indicates the position of the monomer for hHv1-ΔC (50 kDa) and the *purple arrow* the monomer for hHv1-ΔNΔC. *B*, the 2D denaturing PAGE of the lanes in gel A. All high molecular weight bands in A collapse to the expected weight of the monomer for each construct. The second less intense higher molecular weight band observed is likely a glycosylated form of the channel.

To further explore the assembly of WT and mutant channels employed in this study, we performed a native protein Western blot. We used the mCitrine-tagged version of the channels to be able to use an anti-GFP antibody to recognize them. As shown in Figure 5*A*, the native non-denatured proteins exist in multimeric states. For the WT channel, Western blot indeed shows a band at ∼120 kDa, the expected weight of the dimer, but there is also a prominent band at a weight corresponding to tetramers or dimers of dimers. Both ΔC and the ΔNΔC-hHv1 channels show bands corresponding to dimers and higher order oligomers, as well as bands near the expected monomer. To make sure that these high molecular weight complexes are formed by association of a single type of protein, *i.e.*, an Hv1 monomer, we excised the entire lanes of the native PAGE and ran a 2D denaturing PAGE. Western blotting of these 2D gels show that all high molecular complexes collapse to near the expected weight of the monomeric construct, indicating that the native proteins detected in Figure 5*A* are formed by subunits of Hv1. This biochemical data is consistent with the FRET observed in the supposedly monomeric constructs and confirms that the N- and C-termini are not strictly necessary to form dimeric channels.

In order to extend the results obtained with hHv1 channels, we explored the oligomerization state of an Hv1 proton channel from a different organism.

We recently cloned and characterized an Hv1 protein from the scleractinian corals *Acropora millepora* and *Acropora palmata* (Anthozoa, scleractinia), which share 34% amino acid sequence identity with hHv1 ((21) and Fig. S2). For these experiments, we centered on the *A. millepora* Hv1, which we call AmHv1. Sequence analysis of AmHv1 indicates that the C-terminus has a larger propensity to form a coiled-coil structure than the human counterpart (21).

These coral-derived proton channels are activated by voltage up to 10 times faster than their human counterpart and for this reason, proton currents are more easily recorded after digital subtraction of linear current components, allowing for a more precise characterization of activation kinetics than for the human ortholog. First, we determined the activation properties of WT AmHv1 with a fused mCitrine FP in the N-terminus and compared them with ΔC channels, also with mCitrine in the N-terminus. WT AmHv1 channels have a pronounced sigmoidal activation time course, similarly to hHv1. To analyze the sigmoidicity in these channels, we employed a different method to that used in Figure 1 for hHv1. Since linear current components can be suitably subtracted using a -p/4 protocol, leaving only the channel-mediated currents, we fitted activation time courses of subtracted currents to an exponential function raised to a power of *n* (Equation 4). This equation encodes the sigmoidal activation in the power *n* of the exponential (22). Since no linear components are present, the estimation of the activation delay from Equation 4 is more reliable, since we can fit the entire time course of the currents, not only the second half of the activation.

**Figure 6:**
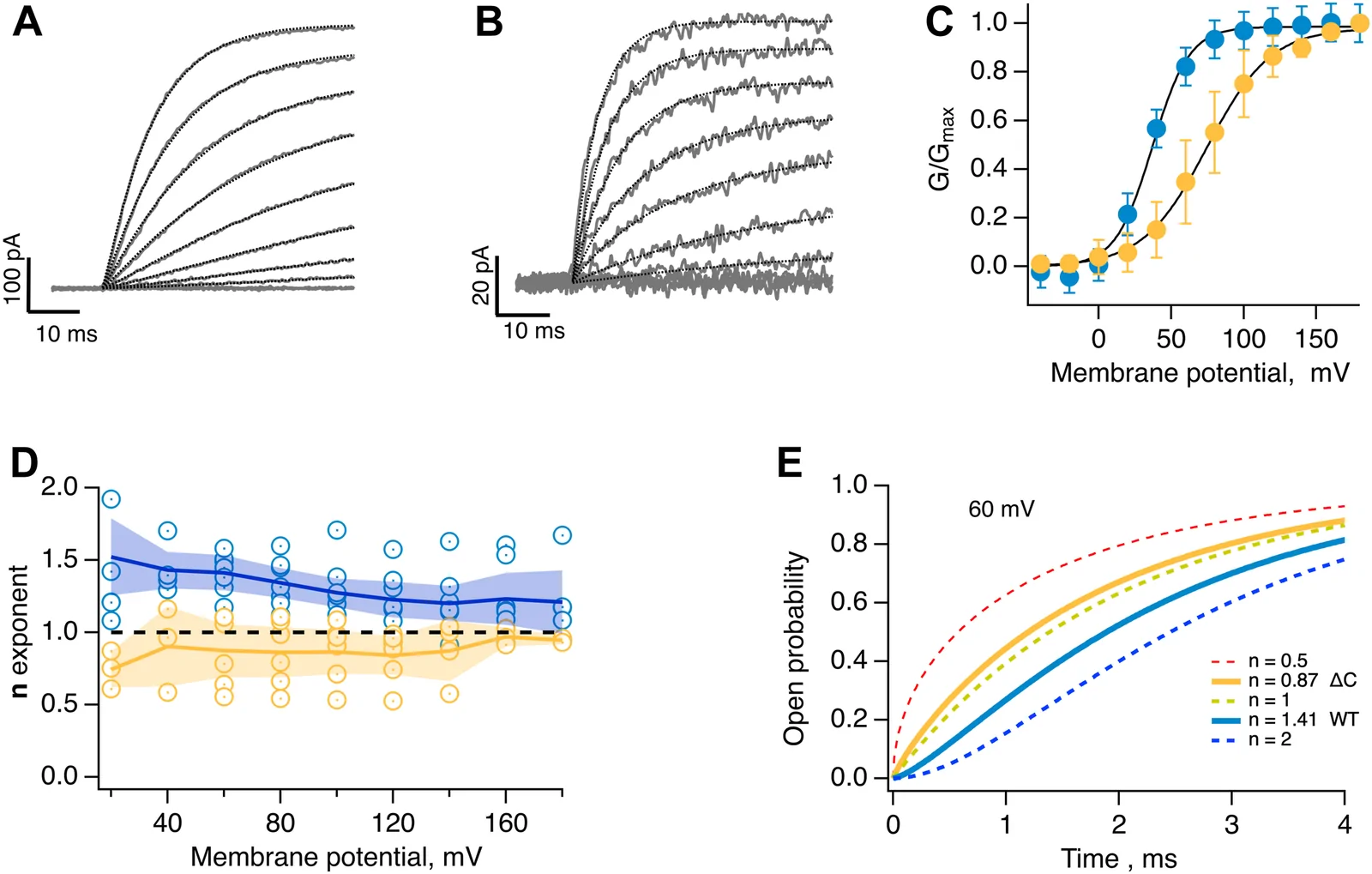
**Sigmoidicity of activation of AmHv1 and AmHv1-ΔC.***A*, mCitrine-WT currents (*gray traces*) are fitted with Equation 4 (*dotted curves*) with an n exponent that varies with voltage. Activation occurs with a delay and with a sigmoidal time course. *B*, currents of mCitrine-ΔC channels (*gray traces*) fitted to Equation 4 as in *A*. Note the near-exponential time course of activation. ΔpH = 1 for both experiment groups. *C*, voltage-dependence of conductance activation of WT (*blue circles*) and ΔC channels (*yellow circles*). Continuous curves are best fits to Equation 2 with parameters: WT, 1.97 e_o_, 37.1 mV; ΔC, 1.14 e_o_, 74.7 mV. *D*, summary of the values of the exponent *n* as a function of voltage derived from fits as in *A* and *B*. *Blue circles* are WT and *yellow circles* are ΔC channels. The *continuous line* is the mean value, and the *shadowed areas* are the SD. *D*, comparison of the activation kinetics as expressed by an exponential function raised to a power *n*. *Dotted curves* are predictions for different values of *n*. *Continuous curves* are the kinetics of an exponential raised to the average power *n* that was estimated from data at 60 mV, for both WT and ΔC channels.

As shown in Figure 6*A*, WT AmHv1 channels activate rapidly and exhibit sigmoidal time course. The currents can be well modeled by Equation 4 with n > 1. Interestingly, n is voltage dependent, being larger at intermediate voltages and approaching a value of ∼1.2 at voltages more positive than 120 mV (Fig. 6*C*). The value of this exponent is similar to what was found in proton currents from human (23). In contrast, the AmHv1-ΔC channels activate almost immediately after a voltage pulse, following exponential activation (*n* = 1). Removal of the C-terminus shifts the activation voltage to more positive voltages (Fig. 6*C*). These results are generally consistent with the effect of deleting the C-terminus on the activation of hHv1 channels (Fig. 1).

**Figure 7:**
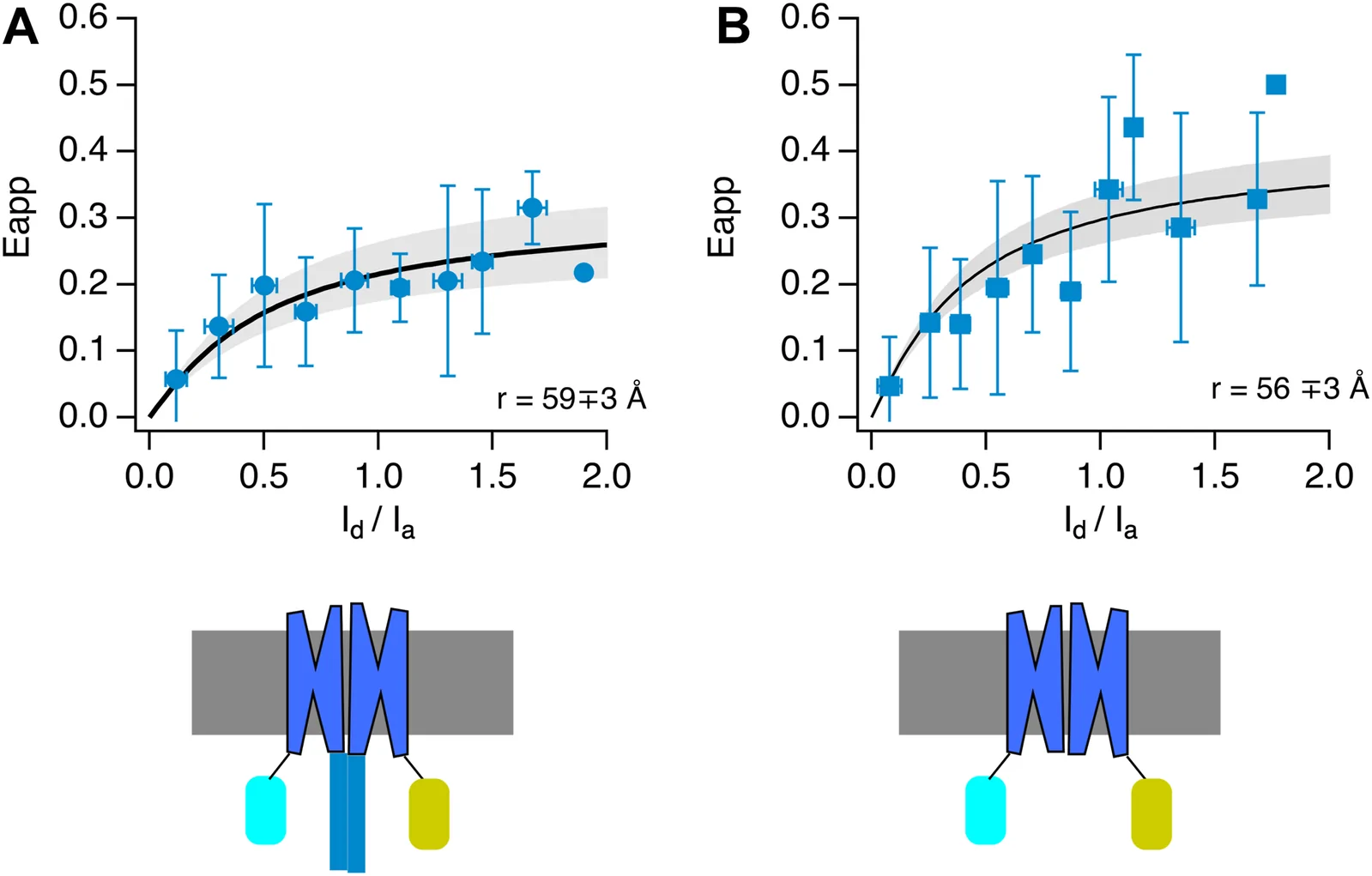
**Stoichiometry of AmHv1 channels estimated by FRET.***A*, WT channels tagged at the N-terminus with mCitrine or Cerulean fluorescent proteins were coexpressed in HEK293 cells. The assembled channels produced robust FRET signals that are consistent with a dimeric stoichiometry. An estimate of the distance r between the FRET pair is shown. *B*, the FRET signal from coexpressed ΔC-mCitrine and mCerulean subunits is also robust, and is consistent with these channels also assembling as dimers, as in the human ortholog, as indicated by the fit (*continuous curve*). Symbols are the mean apparent FRET and the error bars are the SD. Data from 155 cells for ΔC and 134 for WT. AmHv1, *Acropora millepora* human Hv1; HEK, human embryonic kidney.

By applying the FRET approach to the AmHv1 channel and its corresponding ΔC mutant, we estimated their oligomerization state. As with the human channel, the FRET efficiency of WT channels tagged with FPs in the N-terminus is high and consistent with a dimer (Fig. 7*A*). Finally, expression of only the ΔC construct also results in high FRET efficiency (Fig. 7*B*), which is consistent with dimerization of channels lacking the C-terminus. These results indicate that as with hHv1, the coral channel can multimerize when the C-terminus is not present.

**Figure 8:**
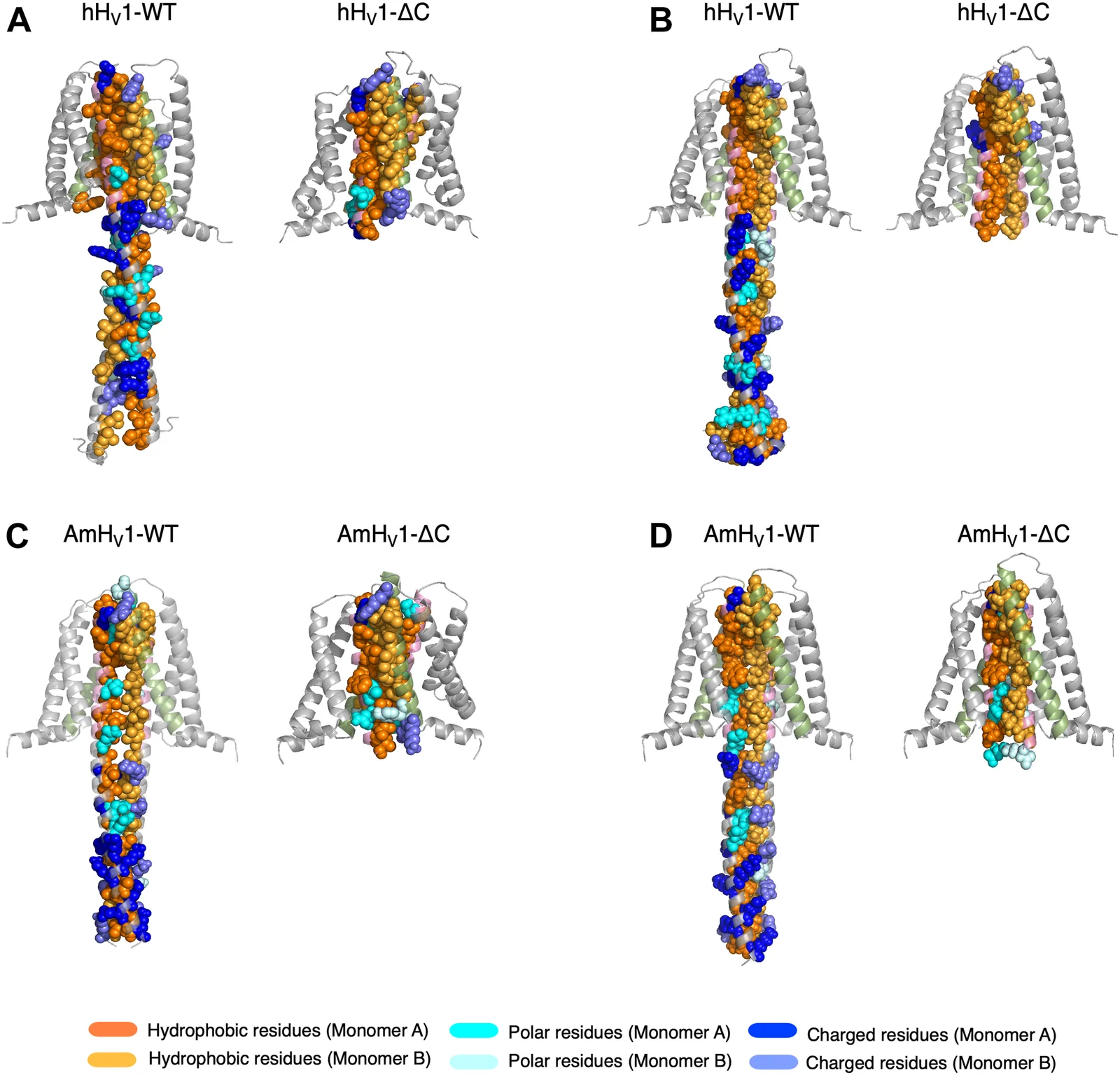
**Wild-type and ΔC human channels can theoretically form dimers.***A*, dimeric assemblies in WT and ΔC channels generated in HADDOCK. In the WT dimer, the main interaction surface is mediated by the S4-S4 and S1-S4 segments, as well as the C-terminal helix. Dimer interaction in ΔC channel is formed by S1-S4 and S4-S4 segments of different subunits. *B*, dimer assemblies of WT and ΔC channels generated using Alphafold2-multimer. In WT channels, the main interaction surface is formed by the S4 segments and the C-terminal helix. The interaction surface in ΔC channels is formed by the S4 segments of different subunits according to the AlphaFold prediction. In addition, some residues of the S1 segments contribute to the interaction surface with the S4 segment. The S1 and S4 segments of each monomeric subunit are depicted in *pink* and *green*, respectively. Interface residues are represented as *spheres*, color-coded as indicated. The segments S2, S3, and the N- and C-terminal regions are in *gray*. *C*, dimeric assemblies of WT and ΔC AmHv1 channels, respectively, generated in HADDOCK. In the WT channels, the primary interaction surface is formed between the S4 segments and the extended C-terminal helix of each subunit. With the participation of some residues of the S1 helix. In the AmHv1-ΔC dimeric assembly, interactions are formed between the S1-S4 and S4-S4 segments of different subunits. *D*, dimer assemblies of WT and ΔC AmHv1 channels, respectively, generated using Alphafold2-multimer. In WT channels the main interaction surface is formed by the S4 segments and the C-terminal helix. The interaction surface in ΔC channels is formed by the S4 segments of different subunits. The S1 and S4 segments of each monomeric subunit are depicted in *pink* and *green*, respectively. Interface residues are represented as *spheres*, color-coded as indicated. The segments S2, S3, and the N- and C-terminal regions are in gray. HADDOCK, High Ambiguity Driven protein-protein DOCKing; AmHv1, *Acropora millepora* human Hv1.

To investigate possible multimerization interfaces of WT and ΔC Hv1 channels, we employed the High Ambiguity Driven protein-protein *DOCKing* (HADDOCK) protein-protein docking algorithm using models of Hv1 generated in AlphaFold2 (Fig. 8). The HADDOCK algorithm predicts WT and ΔC human dimers, in which the interaction is mediated by the S4-S4 and S1-S4 segments (Fig. 8*A* and Table S1). Interface hydrophobic residues found in the S1 and S4 segments include V103, I106, V110, L117, L120, I121 and L124 in S1 and I196, L199, L200, I202, L203, V206 and I217 in S4. From these residues only L120, I196 and L199 were not selected as active during the docking process. The other residues are consistent with the interface residues of the S1 and S4 segments of the CiHv1 and mHv1 dimer interface identified through mutagenesis experiments (11, 24). HADDOCK also predicts Q102 on the S1 segment as part of the interface.

Since HADDOCK docking is guided by selecting initial interacting residues, we were interested in nonbiased docking provided by AlphaFold2. We obtained WT and ΔC Hv1 dimeric channels that were predicted by the AlphaFold2-multimer algorithm (Fig. 8*B*). Interestingly, the hHv1-WT and hHv1-ΔC dimers modeled by AlphaFold2-multimer, without a template, contain the S4-S4 interaction surface as a major contributor to the assembly between subunits. Residues, A113, L117, I121, L124, and K125 in the S1 of each subunit are at the interface of both versions of the dimers (Fig. 8*B*). This interacting surface maintains L117, I121 and L124 residues found in the S1 surface of the HADDOCK prediction and also include other S4 residues (L206, A210 and I217) described in previous experimental data (24).

Residues of the C-terminus interface in the WT dimers of both HADDOCK and AlphaFold2-multimer (Fig. 8, *A* and *B*) include E225, Q227, L228, L231, K232, M234, N235, L238, A239, K241, I242, L245, E246, S248, C249,K252, E253, E255, I256, L259, N260, L262, L263, and H266, which correspond to those identified in the coiled-coil interaction described by previous structural analyses (16, 25), although a complete coiled-coil interaction is not formed in the hHv1-WT predicted by HADDOCK. The coiled-coil interaction between the C-terminal regions of hHv1-WT predicted by AlphaFold2-multimer extends to L268, E271 and V272 residues.

Using a similar approach, we extended our analysis to AmHv1 channel multimerization. AmHv1-WT and AmHv1-ΔC dimers predicted by HADDOCK and AlphaFold2-multimer conserved the S1-S4 and S4-S4 interacting surfaces (Fig. 8*C*), similar to the hHv1 channels. Predictions of the AmHv1-WT and the AmHv1-ΔC channels by AlphaFold2-multimer show fewer residues of the S1 segment (L84, L88, and G91) at the interface interaction (Fig. 8*D*). Most of the residues identified at the interface of each segment correspond to those specified during the docking process in HADDOCK, for S1 (Y67, I81, V85, L88, and R89) and S4 (D160, S163 L164, V166, L170, V173, T174, V177, N178, I182, and Q185. Moreover, the S4 segment residues are conserved in the interaction surfaces of the dimers predicted by AlphaFold2-multimer (Fig. 8*D*). The residues E189, K191, V192, V195, M196, E198, N199, L202, Q203, E205, L206, L209, K210, K212, C213, L216, E217, E219, L220, L223, and K224 of the C-terminal regions are conserved in the interaction surface of both predictions of AmHv1-WT channels and equivalent to those identified in structural analyses (16, 25). The modeling results are not meant to be definitive descriptions of the interaction of Hv1 monomers, and should be taken only as a starting point. However, they help our argument that even in the absence of the C-terminal interactions, Hv1 channels from two very different species can form stable dimers through the interaction of transmembrane segments, as borne out of multiple experimental results.

## Discussion

It is generally accepted that the functional form of Hv1 voltage-gated proton channels is a dimer. There are several lines of evidence supporting this interpretation. FRET measurements of channels tagged at external cysteines with organic fluorophores, showed FRET efficiency consistent with interaction between subunits (10, 26). Single-molecule photobleaching experiments indicated a major proportion of two-step bleaching events, consistent with a major population of channels with two fluorescent proteins (12). Biochemical experiments show the presence of multimers consistent with dimers in non-denaturing protein gels (10). The sequence of the C-terminus of Hv1 channels provides a clue to the possible origin of the dimeric structure, since it is predicted to be an alpha helix containing motifs that are common in coiled-coil helices (13).

The hypothesis that a coiled-coil interaction mediated by the C-terminus has received significant support. Deletion of the C-terminus in both human and truncated Hv1 isoforms, yields channels with faster activation and smaller apparent gating charge, which has been interpreted as a sign of cooperativity between the two subunits, possibly mediated by the C-termini interaction. A FRET experiment has been carried out previously in C-terminus-deleted channels (ΔC) that showed complete disappearance of FRET (10), consistent with lack of assembly of the dimer. These previous results have led to the general assumption that ΔC channels are monomers and that some properties of dimers, like the gating charge or the single-channel conductance, are simply the sum of those of monomers.

However, here we present several results that are not consistent with that interpretation. First, patch-fluorometry indicates that the conductance of the ΔC human channel is not simply half that of a WT channel. Our experiments show that for the same expression level, which is proportional to fluorescence intensity, the ΔC channel produces less current. This can be explained if deletion of the C-terminus reduces the single-channel conductance and/or the open probability. Direct measurement of these variables is unfortunately experimentally very hard using single-channel recordings given that the conductance of Hv1is estimated to be exceedingly small.

As previously published, deletion of the C-terminus eliminates sigmoidal activation, and this phenomenology has been explained as a loss of cooperativity because dimers can no longer form. However, if dimeric channels without sigmoidal activation can be assembled by Hv1-ΔC how can the exponential activation of Hv1-ΔC be explained? One possibility is that in the absence of the coiled-coil interaction, early transitions which might be stabilized by this interaction become faster and the gating transition occurring before channel opening becomes rate-limiting, leading to activation appearing mono-exponential. This is clearly illustrated by simulations using a four-transition kinetic model (Fig. S3). The estimated gating charge from the ΔC channel is not half of WT channel, as it would be expected from a monomeric channel, instead, it is reduced by one third, a similar reduction is observed when the first arginine residue of the S4 segment is neutralized (6).

The most surprising result, derived from FRET measurements and native protein western blots, is that deletion of the C-terminal helix (ΔC channels) does not preclude formation of multimeric channels, most likely dimers. One possible complication of FRET experiments employing fluorescent proteins as donor/acceptor pairs, is the possibility of interaction between the introduced tags. For this reason, we have employed monomerized versions of cerulean and mCitrine, also, the estimated separation between donor/acceptor is larger than what can be expected from a fluorescent proteins dimer (∼22 Å) (27). While monomeric fluorescent proteins largely reduce the possibility of unspecific multimerization, the local concentration of fluorescent proteins tagged membrane proteins can be very large. The density of channels can be estimated from the data in Figure 2. Simplifying the geometry of the patch as a hemisphere with average radius 10 μm, measured directly from our images, the average area of patches is 628 μm^2^. The single-channel conductance of hHv1 in eosinophils has been estimated to be 38 fS (28). This means that the channel density in our patches expressing the largest currents (2 nA) is approximately 1034 channels/μm^2^. If, as a first approximation, channels were disposed on a regular square lattice, the x, y separation between channels is approximately 300 Å, which is almost five times the Ro value for FRET between mCitrine and mCerulean. For HEK293 cells, the average current density we measure is 200 pA/pF, which is equivalent to ∼660 channels/μm^2^, which translates to an even larger separation between channels, again, considering organization in a regular lattice. This means that, although the channel density in oocytes can be high, the channels are separated by an average distance that is larger than what is required to produce FRET or homo-FRET.

This calculation is consistent with a control experiment and previous experiments in which we have shown that coexpression at high density of tagged Hv1 with other tagged ion channels does not yield a measurable FRET signal, supporting the idea that fluorescent proteins do not induce nonspecific multimerization and that FRET is the consequence of dimer formation between Hv1 or Hv1-ΔC channels (29).

Further evidence is provided by channels lacking both the N- and C-termini, ΔNΔC-hHv1, which readily produce FRET that is consistent with multimeric assembly, likely mediated only by the transmembrane domains.

The effect of deleting the C-terminus in an Hv1 channel cloned from a coral, is similar to the human channel. The conductance *versus* voltage curve is shifted to positive voltages by ∼30 mV and currents activation loses sigmoidicity. Moreover, similar FRET experiments carried out in this Hv1 channel, lead to comparable results, showing that ΔC channels can form homodimers. The similarity of results from this variant and hHv1 channels, suggest that oligomerization in the absence of the coiled-coil is evolutionarily preserved.

It was early recognized that cysteines introduced in the S1 of hHv1 could form disulfide bridges, which requires close proximity between them and suggested that the transmembrane domain could also form an inter-subunit interaction region (13). Recently, another interaction between transmembrane domains S4 and S1 has been characterized in CiHv1 (11).

The prevailing hypothesis is that coiled-coil removal abolishes the inter-subunit cooperativity that would normally modulate gating in the WT dimeric complex (15); however, loss of cooperativity and shift of the voltage dependent gating can be induced by a single point mutation in the S2 segment (E153) (17). Furthermore, the mutation of three residues in the coiled-coiled disrupts cooperative gating without disruption of the dimer (16).

This shows that the role of the C-terminus in channel gating is more complex than simply dimerization, as our patch-fluorometry results indicate. In this line, co-immunoprecipitation (co-IP) of differentially epitope-tagged Hv1 channels is reduced, but not completely prevented by C-terminal truncation (10), suggesting that truncated subunits retain the capacity to physically interact.

Finally, our docking predictions suggest that ΔC channels retain the capacity to form stable dimers and that some of these interactions are the same as those observed in the WT dimers obtained by docking. The fact that similar interfaces are obtained by guided or unguided docking methods and that docking results are compatible with experimentally observed interactions in WT dimers (11), suggests that these interactions are robust. Finally, it has recently been reported that the CiVSP phosphatase, which is a VSD thought to be monomeric, can form dimers through interactions of its transmembrane domains (30), similar to the ΔC and ΔNΔC dimers reported here.

## Experimental procedures

### Channels and constructs

hHv1 fused at the C-terminus to mCitrine or mCerulean fluorescent proteins are cloned in pCDNA6.2-DEST and were a gift of Dr Justin Taraska at NIH. These constructs were used to produce fusions of mCitrine or mCerulean at the N- or C-termini and the corresponding C-terminus deletions (ΔC) or combined C- and N-termini deletions (ΔCΔN). Fluorescent proteins were fussed with Hv1 with a flexible linker consisting of four glycine residues. The rat TRPV1 ion channel tagged in the C-terminus with YFP in the pCDNA3 vector was used for control experiments.

For patch-clamp fluorometry, we used hHv1 fused to monomeric mVenus fluorescent protein in the N-terminus and cloned in pBSTA vector, which allows mRNA synthesis for expression in oocytes. The AmHv1 was cloned from total mRNA obtained from a fragment of coral from a commercial marine aquarium (21). Deletions and fusions with mCitrine or mCerulean were produced by overlap PCR and the final products cloned in the pCDNA3.1 expression vector. Fluorescent proteins were also fused to AmHv1 with a four-glycine linker. All constructs were confirmed to be correct by automatic sequencing at the Molecular Biology Unit of the Institute for Cellular Physiology at UNAM).

FRET experiments were carried out in HEK293. HEK293 cells were grown at 37 °C in a humidified atmosphere of 95% air and 5% CO2, cells were transfected using JetPei (Polyplus Transfection) following previously described methods (29) with plasmids containing appropriate constructs; experiments were performed 3 or 4 days after transfection.

*Xenopus* frog’s husbandry and surgery for oocyte extraction were carried out according to NIH guidelines (31). Oocytes were defolliculated and incubated at 18 °C in standard ND96 solution and injected with 36 nl of mRNA (∼1 μg/ml) 1 day after harvesting using a Nanostepper (Drummond Scientific) with micropipettes of ∼20-μm-diameter opening. Experiments were performed 3 to 5 days after injection.

### Electrophysiology

hHv1 channels were expressed in *Xenopus* oocytes following previously published methods (20). AmHv1 channels were expressed in HEK293 cells. Macroscopic currents were recorded from inside-out patches, the extracellular (pipette) solution contained (in mM): 80 tetramethylammonium and methanesulfonic acid, 100 buffer 4-(2-hydroxyethyl)-1-piperazineethanesulfonic acid (Hepes): pH 7.5, 2 CaCl_2_, 2 MgCl_2_, and pH-adjusted N-methyl-d-glucamine/tetramethylammonium hydroxide and HCl. The intracellular bath solution was: 80 tetramethylammonium and methanesulfonic acid, 100 buffer MES, 1 ethylene glycol tetraacetic acid (EGTA), and pH-adjusted N-methyl-d-glucamine/tetramethylammonium hydroxide and HCl to 6.5. Macroscopic proton currents were sampled at 10 kHz and filtered at 2 kHz with an Axopatch 200B amplifier and an Instrutech ITC-18 (HEKA Elektronik) using Patchmaster software (HEKA Elektronik). Membrane patches were initially held at −90 mV for 10 ms, and the voltage was then stepped to positive voltages in steps of 10 or 20 mV. For the much faster-activating AmHv1, pulse duration was 300 ms and linear current components were subtracted using a -p/4 protocol from a subtraction holding potential of −120 mV. Four subtraction pulses were averaged and there was a delay of 200 ms between each. For recordings of hHv1, no subtraction was done. All recordings were performed at room temperature (∼22 °C).

### Data analysis

Conductance was calculated from I-V relations assuming ohmic microscopic currents, according to:

(1) $$I\left(V\right)=g\cdot \left(V-V_{rev}\right)$$

The normalized conductance-voltage (G-V) relations were fit to a Boltzmann function according to Equation 2.

(2) $$\frac{g}{g_{\max}}=\frac{1}{1+\exp\left(\frac{q\left(V-V_{0.5}\right)}{K_{B}T}\right)}$$

Here, *V*_*0.5*_ is the voltage at which *g/g*_*max*_ = 0.5, *q* is the apparent gating charge (in elementary charges, *e*_*o*_) and *K*_*B*_ is the Boltzmann constant and *T* temperature in Kelvin (22 °C).

The second half of macroscopic currents from hHv1 were fit to the equation:

(3) $$I\left(t\right)=I_{ss}\cdot \left(1-e^{\left(\frac{-\left(t-\delta \right)}{\tau }\right)}\right)$$

Where *I*_*ss*_ is the amplitude of the current at steady-state, *δ* is the delay of the exponential with respect to the start of the voltage pulse and *τ* is the time constant, both with units of ms.

The degree of sigmoidicity of activation for the coral Hv1 channels, was estimated from the value of the exponent *n* in the following function:

(4) $$I\left(t\right)=I_{ss}\cdot {\left(1-e^{\left(-\frac{t}{\tau }\right)}\right)}^{n}$$

### Patch-clamp fluorometry

Currents and patch fluorescence were measured simultaneously in the same setup used for FRET measurements (see below). mVenus fluorescent protein was excited by the 488 nm line of an Ar-Ion laser, selected with a 488/5 nm laser band-pass filter (Chroma) and fluorescence collected with an appropriate dichroic mirror (Chroma). Emitted light was recorded by a LucaR or Ixon 867emCCD camera and a 530/20 nm emission filter. Patch fluorescence was measured employing a region of interest that only enclosed the part of the patch that was not in contact with the patch pipette.

### FRET measurements

FRET experiments were carried out in HEK293 cells. The apparent FRET efficiency (*E*_*app*_) between mCitrine and mCerulean -containing constructs was measured by the spectra-FRET method (29, 32) in a home-modified TE-2000U inverted epifluorescense microscope (Nikon). The excitation light source was an Ar-Ion laser (Spectra-Physics) mainly producing light at 458, 488 and 514 nm; the laser beam is focused and then collimated using a 3 mm ball lens and a 50 mm focal length planoconvex lens. Collimated light is steered with a mirror and then focused into the objective back focal plane by a 300 mm focal length achromatic lens.

Cells were imaged with a Nikon 60X oil immersion objective (numerical aperture 1.4). The detection arm of the microscope is coupled to a spectrograph (Acton Instruments) and an EMCCD camera (LucaR or Ixon 867, Andor) controlled by Micromanager software (33).

To obtain the fluorescence emission spectra, the spectrograph entrance slit was partially closed to image a small area of a cell. The emission light was reflected on the grating in the spectrograph to obtain a spectral image, which is then collected by the camera. From this image, an emission spectrum of the membrane area was obtained using a line scan along the wavelength axis.

Emission spectra of cells expressing mCerulean-only constructs were recorded with 458 nm excitation. Direct excitation (*ratioA*_*o*_) of mCitrine was determined as the ratio of the emission spectra observed by exciting the mCitrine at 458 nm and 488 nm. These two measurements help account for cross-talk between mCerulean excitation and mCitrine emission.

Cells cotransfected with a mCerulean and mCitrine constructs are excited at 458 nm, the emission spectrum shows two peaks, the first one at ∼488 nm is the emission of the mCerulean protein and the second at ∼525 nm is the emission of mCitrine. This second peak contains three components, the first due to bleed-thru of mCerulean, the second to direct excitation of mCitrine at 458 nm and the third due to FRET with mCerulean. To quantify the FRET efficiency, the mCerulean -only spectrum, was scaled to the first peak of the emission spectrum and then subtracted, leaving only the mCitrine spectrum, containing two components, the direct excitation at 458 nm and the FRET with mCerulean. Apparent FRET efficiency, *E*_*app*_, is obtained as the difference: *E*_*app*_ = *ratioA – ratioA*_*o*_, where *ratioA* is the ratio between the subtracted mCitrine spectra and a third spectrum obtained by exciting at 488 nm. From the emission spectrum for each cell, the fluorescence intensities of donor (I_d_) and acceptor (I_a_) were obtained as the peak intensity of the mCerulean spectrum and the peak intensity of mCitrine. Different values of I_d_ and I_a_ were attained by varying the expression levels of donor and acceptor by transfecting different ratios of the donor and acceptor DNA.

### Quantifying FRET efficiency

To quantify efficiency of FRET and relate it to channel stoichiometry, we used a model described in detail elsewhere (29). Briefly, the model predicts the apparent efficiency of FRET for *n*_*d*_ donors and *n*_*a*_ acceptors depending on the stoichiometry of assembly, it takes into account the probability of appearance of the different populations depending on the number of donors and acceptors present.

The general equation describing the FRET efficiency for a channel with arbitrary stoichiometry formed by *T* subunits is:

(5) $$E_{app}=\frac{\sum_{n_{d}=0}^{T}\left(T-n_{d}\right)P_{n_{d}\left(T-n_{d}\right)}E_{n_{d}\left(T-n_{d}\right)}}{\sum_{n_{d}=0}^{T}\left(T-n_{d}\right)P_{n_{d}\left(T-n_{d}\right)}}$$

Here *T* is the number of subunits, *n*_*d*_ is the number of donors, *(T-n*_*d*_*)* is the number of acceptors, *P*_*nd(T-nd)*_ is the probability of occurrence of a channel with *n*_*d*_ donors and *(T-n*_*d*_*)* acceptors, and *E*_*nd(T-nd)*_ is the intrinsic FRET efficiency of the same channel, calculated from a geometric model of the assembled channel, with fluorophores separated by distance *r* (34).

The fluorescence intensity ratio *Fr* is given by the ratio of total donor intensity to total acceptor intensity:

(6) $$Fr=\frac{I_{d}}{I_{a}}\cdot \frac{S_{d}}{S_{a}}$$

Where:

$$I_{d}=\sum_{n_{d}=0}^{T}n_{d}P_{n_{d}\left(T-n_{d}\right)}\left(1-E_{n_{d}\left(T-n_{d}\right)}\right)$$

$$I_{a}=\sum_{n_{d}=0}^{T}\left(T-n_{d}\right)P_{n_{d}\left(T-n_{d}\right)}\left(1+E_{n_{d}\left(T-n_{d}\right)}\right)\frac{\varepsilon_{d}}{\varepsilon_{a}}$$

Here, ε_*d*_ and ε_*a*_ are the donor and acceptor molar extinction coefficients at the donor excitation wavelength and *S*_*a*_ and *S*_*d*_ are the detection factors for donor and acceptors, respectively.

The predictions for the efficiency of FRET for a given model are compared against data by plotting Equation 5 as a function of Equation 6.

Data analysis and modeling were done within Igor Pro v.8 (Wavemetrics). All summary data are presented as mean ± standard deviation (sd).

### Protein-protein docking

Monomeric structures of WT hHv1 and AmHv1 lacking the N-terminus (amino acids 86–273 and 50–226, respectively) and the ΔC version lacking both N- and C-terminal regions (amino acids 86–221 and 50–185, respectively) were predicted using AlphaFold2-Colab (35) with the default settings and without templates. We selected the top-ranked models with the following pLDDT values: hHv1: 79.9; hHv1-ΔC: 77; AmHv1:79.3; AmHv1-ΔC; 75.8. Next, we performed docking of the modeled Hv1 monomers in the HADDOCK 2.4 server (36). We selected active residues for docking based on equivalent residues in transmembrane segments S1 and S4 from previous experimental data of (11, 24). For the C-terminus, active residues were selected from the interface residues identified in the structural analysis of the C terminus coiled-coil interactions (16, 25). We obtained WT and ΔC dimeric channels and selected as a final dimer the dockings with best HADDOCK score (Table S1).

Dimeric WT, ΔC, and mixed channels were also generated in Alphafold2-mutimer (37) with the default settings and no templates. The quality of all generated models was evaluated using the Molprobity server (38) (Table S2). When necessary, models were energy minimized with 50 steps of the steepest descent (SD) algorithm, fixing all heavy atoms, followed by 50 steps of SD of all atoms, and 50 steps of conjugate gradient of all atoms to eliminate bad contacts using the program CHARMM (v.46) and the Charmm36 force field (39, 40). All the predicted dimers were uploaded to PRODIGY server (41) to predict binding affinity (ΔG) expressed in kcal⋅mol^-1^. The PRODIGY method uses structural and physicochemical properties from the protein-protein interface to compute binding affinity, which includes number of interfacial contacts, noninteracting surface area, number of hydrogen bonds and salt bridges. Two residues are defined in contact if any of their heavy atoms are within a distance of 5.5 Å (41).

### Western blotting

#### Native electrophoresis

The hrCN3-PAGE was performed as described (42); protein samples (30 μg per lane) were applied to a 4 to 14% acrylamide gradient gel. The anode buffer contained 25 mM imidazole/HCl, pH 7.0; the cathode buffer was 50 mM Tricine, 7.5 mM imidazole, pH 7.0, 0.01% n-Dodecyl-β-D-maltoside, and 0.05% deoxycholate. Electrophoresis was performed at 4 °C, and the voltage was set to 35 V for 10 h and stopped when the sharp line of the Ponceau S dye approached the gel front. The molecular weight was determined using the Native Mark Protein Std (Invitrogen, Thermo Fisher Scientific, cat: 57030) as standards.

#### 2D-Tricine-SDS polyacrylamide gel electrophoresis (2D-T-SDS-PAGE)

The 2D-Tricine-SDS polyacrylamide gel electrophoresis (2D-T-SDS-PAGE) was performed according to (43). Proteins from a native hrCN3-PAGE gel strip were separated by 2D-T-SDS-PAGE on a 10% polyacrylamide gel under denaturing conditions. The anode buffer contained 100 mM Tris, 22.5 mM HCl, pH 8.9. The cathode buffer was 100 mM Tris-HCl, 100 mM Tricine, 0.1% SDS. After the run, the proteins were stained with Coomassie (40% methanol, 7% acetic acid, and 0.25% Coomassie© Brilliant Blue R-125). The molecular weight was determined using Precision Plus Protein Standards Dual Color (Bio-Rad, cat: 161–0374) or Unstained (Bio-Rad, cat: 161-0363).

#### Semidry protein electrotransfer

The hr3CN-PAGE gel was equilibrated in 20 ml of Tricine-SDS-PAGE cathode buffer (see above) for 6 h under constant orbital agitation with changes every 30 min. Then the gel was incubated for 60 min in 20 ml of electrotransfer solution (192 mM glycine, 25 mM Tris-HCl, and 20% methanol) under constant agitation. Finally, the proteins were electrotransferred onto polyvinylidine difluoride membranes for 60 min at 25 V. The 2D-T-SDS-PAGE gel was directly incubated in the electrotransfer solution for 10 min and continued with the transfer protocol. The membrane was blocked with 5% low-fat milk in 0.1% TBS-Tween for 1 h at RT. Citrine-fused constructs were detected with the help of anti-GFP primary antibody (Invitrogen, MA5-15256).

The primary antibody was used at a dilution of 1:3000 and incubated overnight at 4 °C. Subsequently, the membrane was incubated with the anti-mouse secondary antibody (Thermo Fisher Scientific, 31,430). The secondary antibody was used at a 1:10,000 dilution, incubating it for 1 h at RT. Finally, the SuperSignal West Pico PLUS kit (Thermo Fisher Scientific, 34577) was used to reveal the protein bands.

#### Simulations of current activation

A five-state kinetic model was employed to simulate time courses of current activation. The coupled system of five differential equations was solved using an in-house written program in Igor Pro 9 implementing the Runge-Kutta algorithm. The voltage dependence of the rate constant for the transition between any state *i* to *j* has the form: $k_{ij}=k_{ij}\left(0\right)\cdot e^{q_{ij}V/K_{B}T}$

*k*_*ij*_*(0)* is the rate constant at 0 mV, *q*_*ij*_ is the charge moved in the transition in elementary charges (e_o_), *V* is the voltage in mV, *K*_*B*_ is the Boltzmann constant and *T* the temperature in Kelvin.

## Data availability

All data are contained within the article. Materials are available from the corresponding author upon request.

## Supporting information

This article contains supporting information.

## Conflict of interest

The authors declare that they have no conflicts of interest with the contents of this article.

## Supporting information

SupplementaryMaterial
