# Supplementary material for: C-terminus–independent multimerization of Hv1 proton channels
Source: J Biol Chem. 2026 Aug 4;302(9):113387. doi: 10.1016/j.jbc.2026.113387 (PMC13546843; doi:10.1016/j.jbc.2026.113387)
Supplement: Supplementary file 1 [file mmc1.docx]

**Supplementary material**

**Supplementary figure 1.** No measurable FRET between highly expressed proteins in the plasma membrane. The TRPV1 channel tagged at the c-terminus with YFP was co-expressed with mCerulean-hHv1 and no detectable FRET was measured. This result indicates that FRET between Hv1 constructs is due to specific interactions and not to multimerization due to fluorescent protein interactions or non-specific interaction between crowded proteins in the membrane.

```
AcHv1               --------------------MIDARTRRSSMDDQLPADELKEMGQPGTTAAVM-------
Human               MATWDEKAVTRRAKVAPAERMSKFLRHFTVVGDDYHAWNINYKKWENEEEEEEEEQPPPT
                                                     .

							  S1
AcHv1               ---------ASTVCLTEDDTECPANKNHREKLNELLHGDKIQYAIVVLVIIDIIIVIAEL
Human               PVSGEEGRAAAPDVAPAPGPAPRAPLDFRGMLRKLFSSHRFQVIIICLVVLDALLVLAEL


					S2			S3
AcHv1               VLDLRAGSEH-HDN-SASHVLHYISIAILSVFMIELLLKIYAMGFTFFKHKMEVFDGFVI
Human               ILDLKIIQPD-KNN-YAAMVFHYMSITILVFFMMEIIFKLFVFRLEFFHHKFEILDAVVV


					  S4
AcHv1               IVSFALDIAFSNEQGGVDGVSLIVLLRLWRVTRIVNGIILSVQMRAEKKVQAVMKENAEL
Human               VVSFILDIVLLFQEHQFEALGLLILLRLWRVARIINGIIISVKTRSERQLLRLKQMNVQL


AcHv1               KTELEQLKSKCAQLESELTTLKQS------------------------------------
Human               AAKIQHLEFSCSEKEQEIERLNKLLRQHGLLGEVN-------------------------
```

**Supplementary figure 2.** Sequence alignment between the *Acropora millepora* Hv1 protein sequence and the human Hv1. Transmembrane segments are indicated by colored text.

**Supplementary figure 3.** Sigmoidal and non-sigmoidal activation can be modulated by a single rate limiting transition. A) An activation model with four transitions can simulate the sigmoidal activation of WT channels and can be used to illustrate how changes in transition kinetics can produce channel with mono exponential activation. B) If early, forward transitions accelerate 10-fold (orange arrows), the opening transition (cyan arrow in A and B) becomes rate limiting and the activation course of currents lose their sigmoidal character. For A) the values at 0 mV of all forward rate constants α= 4 s^-1^, all backward rate constants β=4 s^-1^ and opening rate constant α’=1 s^-1^. For B) α= 100 s^-1^, β=4 s^-1^ and α’=1 s^-1^. The charge associated with each transition is 0.5 e_o_.

| Table S1. Structural metrics of Hv1 dimers predicted in Haddock2.4 | | | | |
| --- | --- | --- | --- | --- |
| **Model** | **Haddock score** | **ΔG (kcal mol^-1^)** | **Interface segments** | **Interface number of contacts within 5.5 Å** |
| hHv1-WT dimer | -149.8 | -8.6 | S1-S4, S4-S4, C-C terminal | 132 |
| hHv1-∆C dimer | -109 | -3.5 | S1-S4, S4-S4 | 82 |
| AmHv1-WT dimer | -138.1 | -10.5 | S1-S4, S4-S4, C-C terminal | 108 |
| AmHv1-∆C dimer | -90.2 | -6.8 | S1-S4, S4-S4 | 73 |

| Table S2. Structural metrics of Hv1 dimers predicted in AlphaFold2-Multimer | | | | |
| --- | --- | --- | --- | --- |
| **Model** | **AlphaFold pLLDT** | **ΔG (kcal mol^-1^)** | **Interface segments** | **Interface number of contacts within 5.5 Å** |
| hHv1-WT dimer | 69.9 | -8.6 | S1-S4, S4-S4, C-C terminal | 134 |
| hHv1-∆C dimer | 64.4 | -3.1 | S1:S4, S4:S4 | 60 |
| AmHv1-WT dimer | 69.4 | -9.3 | S1-S4, S4-S4, C-C terminal | 110 |
| AmHv1-∆C dimer | 65.8 | -4.0 | S1:S4, S4:S4 | 43 |

**Supplementary tables.** Parameters were calculated as explained in the methods section. The number of contacts is high because each residue can have more than one contact within 5.5 Å. HADDOCK score is calculated such that a lower value models are better ranked. AlphaFold2 pLLDT score ranges from 0-100. Scores higher than 70 are considered good backbone predictions. All models were energy minimized before analysis.
